# Supplementary material for: PEPE: scalable extraction of multi-modal protein language model representations
Source: Bioinformatics. 2026 Jun 12;42(6):btag375. doi: 10.1093/bioinformatics/btag375 (PMC13326402; doi:10.1093/bioinformatics/btag375)
Supplement: btag375_Supplementary_Data [file btag375_supplementary_data.zip › Supplementary Material.docx]

# Supplementary Material

# Supplementary Figures


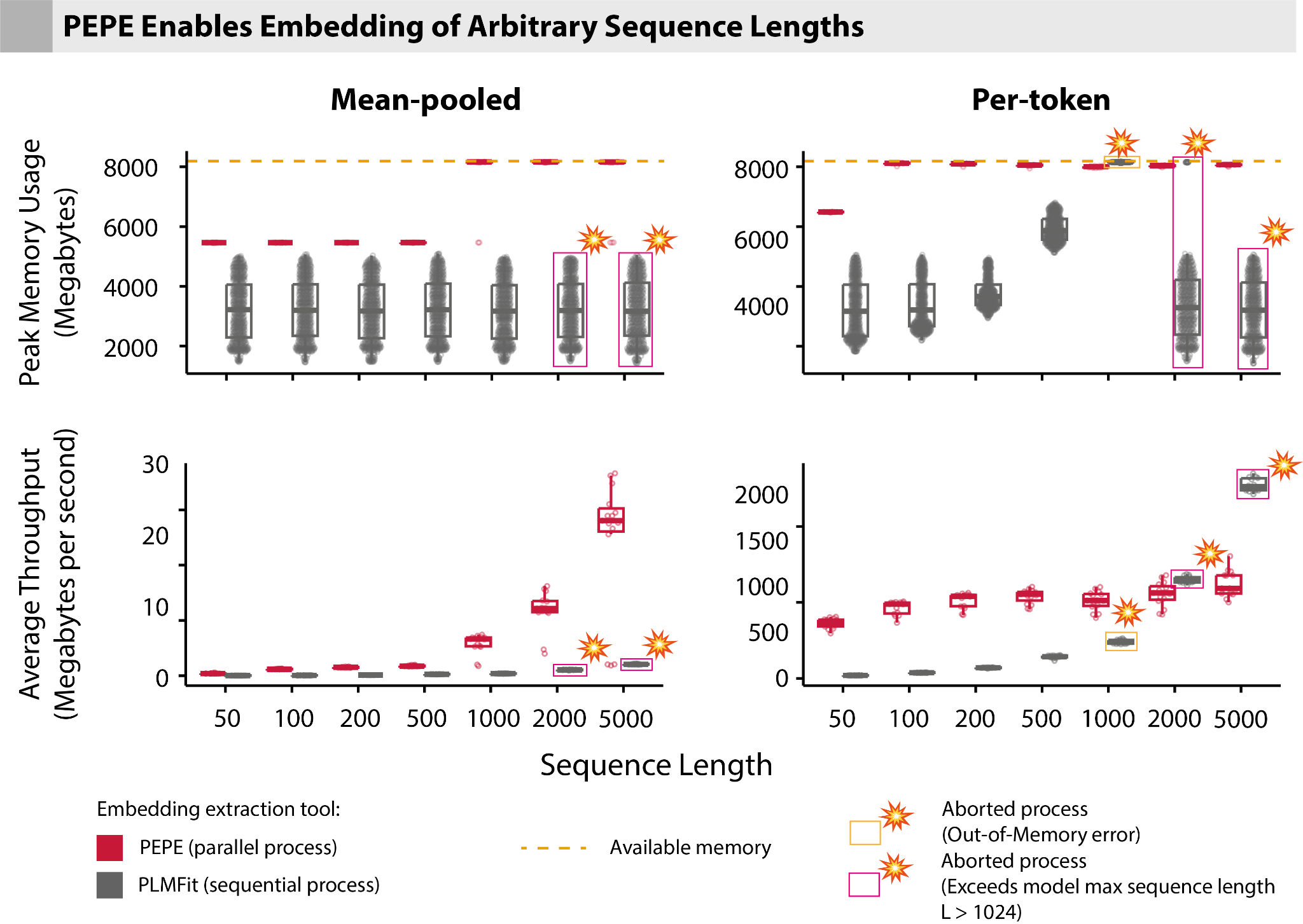


**Supplementary Figure 1: PEPE enables embedding of arbitrary sequence lengths.** Comparison of peak memory usage (upper panels) and average throughput (lower panels) of embedding extraction between PEPE and PLMFit (SOTA). Sets of 1000 random amino acid (AA) sequences with sequence lengths ranging from 50 to 2000 were generated. Both programs were instructed to extract multiple embedding modes (per-token and mean-pooled) from all 33 embedding layers of the ESM-2 model. Per-token embeddings are the unreduced output of the embedding layers, while mean-pooled embeddings are averaged across token-dimensions. All commands were run as SLURM jobs and limited to 1 CPU core, 8192MB of CPU memory (yellow dashed line), and 1 NVIDIA A100 GPU. PEPE’s “Multi-Mode Extraction” was used to extract all 33 layers in a single run, while for the equivalent output, PLMFit’s sequential process requires a repeated run for each layer. Data points in yellow rectangles indicate aborted processes due to out-of-memory errors while magenta rectangles indicate aborted processes due to exceeding ESM-2’s maximum input sequence length of 1022 (plus two special flanking tokens). All measurements were repeated 15 times (Table 3).
