# Supplementary figures and images for: PEPE: scalable extraction of multi-modal protein language model representations
Source: Bioinformatics. 2026 Jun 12;42(6):btag375. doi: 10.1093/bioinformatics/btag375 (PMC13326402; doi:10.1093/bioinformatics/btag375)

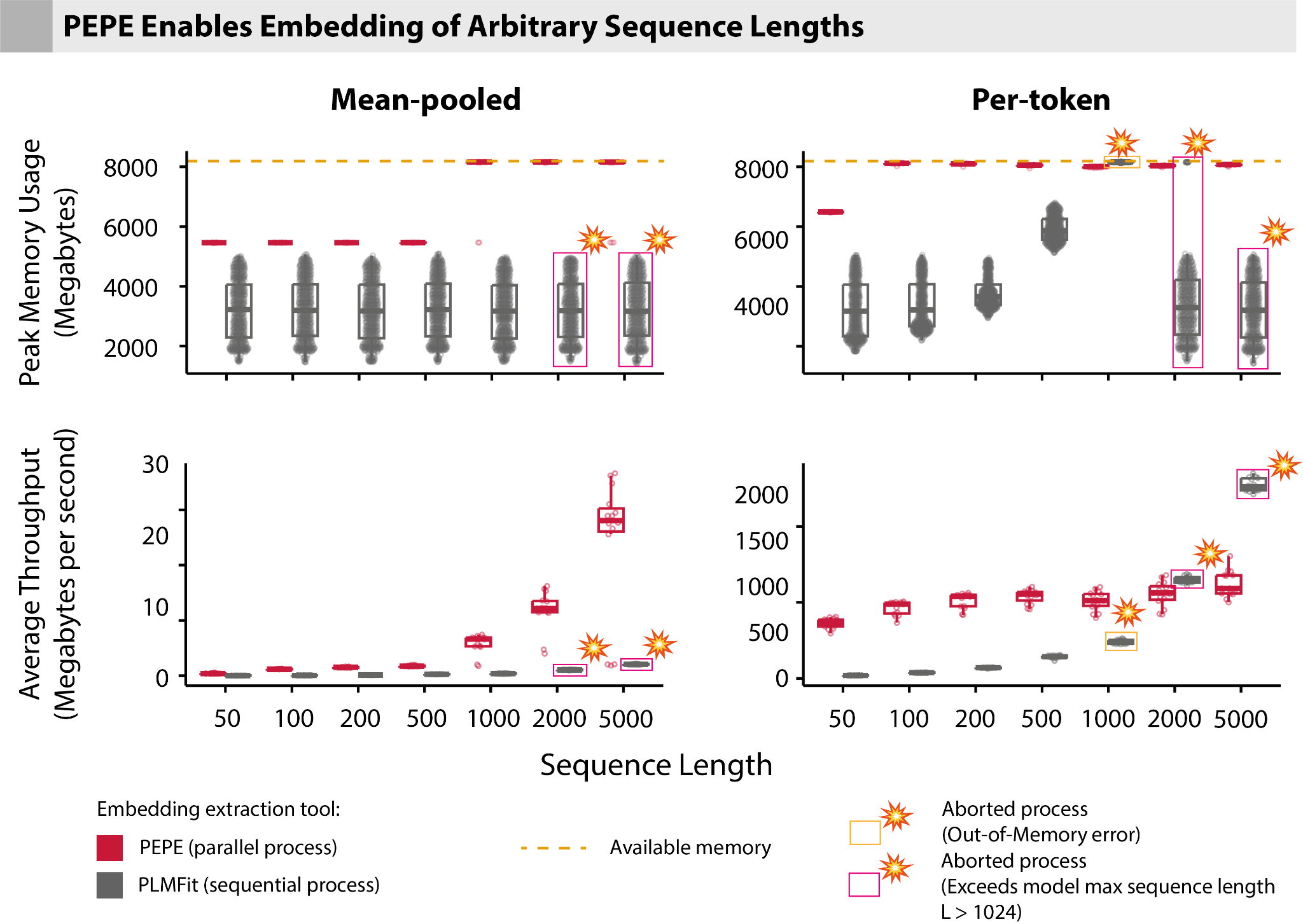

Supplement: btag375_Supplementary_Data [file btag375_supplementary_data.zip › supp_figure1.png]
